# Nogo receptor 1 is expressed by nearly all retinal ganglion cells

**DOI:** 10.1371/journal.pone.0196565

**Published:** 2018-05-16

**Authors:** Alexander M. Solomon, Teleza Westbrook, Greg D. Field, Aaron W. McGee

**Affiliations:** 1 Developmental Neuroscience Program, Saban Research Institute, Children’s Hospital Los Angeles, Department of Pediatrics, Keck School of Medicine, University of Southern California, Los Angeles, California, United States of America; 2 Department of Neurobiology, School of Medicine, Duke University, Durham, North Carolina, United States of America; 3 Department of Anatomical Sciences and Neurobiology, University of Louisville, Louisville, Kentucky, United States of America; National Eye Centre, UNITED STATES

## Abstract

A variety of conditions ranging from glaucoma to blunt force trauma lead to optic nerve atrophy. Identifying signaling pathways for stimulating axon growth in the optic nerve may lead to treatments for these pathologies. Inhibiting signaling by the nogo-66 receptor 1 (NgR1) promotes the re-extension of axons following a crush injury to the optic nerve, and while NgR1 mRNA and protein expression are observed in the retinal ganglion cell (RGC) layer and inner nuclear layer, which retinal cell types express NgR1 remains unknown. Here we determine the expression pattern of NgR1 in the mouse retina by co-labeling neurons with characterized markers of specific retinal neurons together with antibodies specific for NgR1 or Green Fluorescent Protein expressed under control of the *ngr1* promoter. We demonstrate that more than 99% of RGCs express NgR1. Thus, inhibiting NgR1 function may ubiquitously promote the regeneration of axons by RGCs. These results provide additional support for the therapeutic potential of NgR1 signaling in reversing optic nerve atrophy.

## Introduction

Damage to the retinal ganglion cell (RGC) axons that comprise the optic nerve (ON) can lead to loss of vision. These axons are affected in several diseases, including ON trauma, compression, ischemia, and glaucoma. Axon regeneration and stimulating neural plasticity within the retina may contribute to reversing the effects of injury to the retina and ON. A variety of intracellular and extracellular factors have been found to affect RGC survival and/or increase axon regeneration. These include genes that alter intrinsic growth state such as Krüppel-like factor (Klf) transcription factors [1,2], the modulators of intraocular inflammation oncomodulin and dectin-1 [3–6], trophic factors including BDNF [7], the cell-intrinsic suppressors of regeneration *pten* [8,9], and cell-extrinsic inhibitors of regeneration that signal through the nogo-66 receptor 1 (NgR1) [10,11].

NgR1 signaling pathway may have therapeutic potential, as it is a point of convergence for several ligands inhibitory to neurite outgrowth *in vitro* including Nogo-A [12], and has been implicated in both neural plasticity and axon regeneration *in vivo*. Nogo-A (RTN4a) is a reticulon protein expressed by both central nervous system neurons and oligodendrocytes [13,14]. Nogo-A, together with myelin associated glycoprotein (MAG) [15], and oligodendrocyte myelin glycoprotein (OMgp) [16], contribute to the characteristic inhibitory nature of central nervous system myelin membranes to neurite outgrowth [17]. NgR1, also known as the reticulon 4 receptor (Rtn4R), is notable in that it binds not only all three of these membrane-associated inhibitors expressed by oligodendrocytes [18,19], but also chondroitin sulfate proteoglycans (CSPGs), a distinct family of extracellular molecules inhibitory to neurite outgrowth [10].

Interestingly, NgR1, Nogo-A, and CSPGs have all been implicated as inhibitors of both recovery of function following CNS injury and neural plasticity in the developing visual system. Deletion of the *ngr1* gene prevents the closure of a critical period for eye dominance in mice [20], whereas digestion of the sugar moieties from CSPGs with chondroitinase ABC restores similar visual plasticity in adult rats [21]. Constitutive *ngr1* mutant (knockout, KO) mice also exhibit spontaneous recovery of visual acuity with the restoration of binocular vision in a murine model of amblyopia [22]. Mice lacking *ngr1*, lacking *nogo-a*, or treated with chABC at the injury site, have also been reported to display greater recovery following spinal cord injury [23–25]. This regulation of axonal regrowth by NgR1 signaling has motivated the investigation of the potential role for NgR1 in promoting the regeneration of RGC axons following ON injury.

Several studies have examined the role of NgR1 in RGC survival and regeneration following injury to the ON. Genetic deletion of NgR1 has been reported to improve axonal regeneration by RGCs following ON crush injury [10,11,26]. Both intravitreal injection of an anti-NgR1 antibody or a rat NgR1 decoy protein increased RGC survival following episcleral and limbal vein photocoagulation [27]. Furthermore, intravitreal injection of a human NgR1 decoy protein into rat eyes increased the number of RGCs with regenerating axons following ON crush injury and improved RGC survival to control levels in a microbead model of glaucoma [28]. Thus, neutralizing NgR1 correlates with elevated axonal regrowth after ON damage and enhanced RGC survival.

To determine the potential scope of neutralizing NgR1 as a therapeutic target, the diversity of retinal cell types that express NgR1 must be determined. Previous immunohistochemical studies have shown that NgR1 is expressed in the ganglion cell layer (GCL), retinal nerve fiber layer (RNFL), and ON in developing and adult rodents [29]. Studies in adult mice reveal that NgR1 is expressed in at least some RGCs via co-labeling with fluorogold and Brn3a [27,30]. However, RGCs comprise at least 30 distinct cell types [31], including relatively sparse RGCs that express melanopsin and represent only 2% of the total population [32]. These types vary in their protein expression, morphology, function, and projection patterns to the brain [33]. Whether NgR1 is expressed in a subset of RGCs or more extensively is not known. If NgR1 is expressed in only a subset of RGC types, this would likely limit the therapeutic potential of inhibiting NgR1 on RGC survival and ON regeneration. Furthermore, localization of NgR1 to the GCL does not specify its expression to RGCs; approximately 60% of the neurons in the GCL of mice are displaced amacrine cells [34]. The relative expression of NgR1 in RGCs versus amacrine cells in the GCL is also unknown.

The aim of this study was to determine the diversity of retinal cell types that express NgR1 in the GCL of the mouse. We employed immunohistochemistry in combination with a genetic reporter line that expresses green fluorescent protein (GFP) under control of the *ngr1* promoter. To determine if expression in the GCL was restricted to RGCs we used the recently described pan-RGC antibody RBPMS [35]. To examine the expression of NgR1 in specific RGC types including ON-OFF directionally sensitive RGCs, ON-sustained and OFF-transient alpha RGCs, and parvalbumin (PV)-positive RGCs, we co-labeled with well-characterized antibodies specific for these RGC types in mouse. Finally, we investigated the prevalence of NgR1 expression in amacrine cells in the GCL by co-labeling with antibodies directed against gamma amino butyric acid (GABA) and choline acetyltransferase (ChAT).

## Methods

### Mice

The conditional mutant *ngr1* mouse line (*ngr1 flx)* has been described [11]. The ER-Cre mouse line, *B6*.Cg-Tg(CAG-cre/Esr1*)5Amc/J, was purchased from the Jackson Laboratory (stock # 004682). Mice of the genotype *ngr1 flx/+* following cre-mediated deletion of *ngr1*, *ngr1* Δ/+, were used for these studies [11]. Recombination of the *ngr1 flx* allele was confirmed by PCR genotyping with custom primers.

Mice were maintained and all experiments were conducted according to protocols approved by the Children’s Hospital Los Angeles and the University of Louisville Institutional Animal Care and Use Committees. Mice were anesthetized by isoflurane inhalation and euthanized by carbon dioxide asphyxiation or cervical dislocation following deep anesthesia in accordance with approved protocols. The Children’s Hospital Los Angeles and University of Louisville Institutional Animal Care and Use Committee specifically approved this study. Protocol number 264–12 and 16716.

### Tissue preparation

The eyes were dissected in refrigerated HyClone Dulbecco’s Modified Eagles Medium (GE Healthcare Life Sciences, Logan, UT). The eyecups were then immersion-fixed in 4% (w/v) paraformaldehyde (PFA) in 0.1 M phosphate buffered saline (PBS), pH 7.4 for 45 minutes to 1 hour and cryoprotected overnight in 30% sucrose. These eyecups were sectioned at 16–20 μm with a Leica cryostat (Leica Microsystems, Buffalo Grove, IL) and mounted onto slides, which were stored at -20°C. For whole-mounted retinas, the sclera was removed and the retina was flattened photoreceptor side down on black filter paper (EMD Millipore Corporation, Bedford, MA). The retina was subsequently immersion-fixed in 4% PFA in 0.1 M PBS for 1 hour. The whole-mounted retina was stored in 0.1 M PBS until processing for immunohistochemistry.

### Immunohistochemistry

Immunohistochemical labeling was performed based on an indirect immunofluorescence method. Retinal sections were incubated in a solution of 10% normal donkey serum (NDS) and 0.5% Triton X-100 in 0.1 M PBS for 1 hour at room temperature. The blocking solution was washed away, and the sections were immediately incubated with primary antibodies in solution (3% NDS and 0.5% Triton X-100 in 0.1 M PBS, pH 7.4) for 12–16 hours at 4°C in a humidified chamber in the dark. Retinal sections were washed in PBS to remove excess primary antibodies and secondary antibodies were applied for 1 hour at room temperature in the dark. After a final wash the sections were cover-slipped with Fluoromount-G (Southern Biotech, Birmingham, AL). Whole-mounted retinas were incubated in the same blocking solution as retinal sections, overnight at 4°C, followed by incubation in primary antibodies (see Table 1) for 5 to 7 days at 4°C. Retinas were rinsed three times for 30 minutes with 0.1 M PBS and incubated with the corresponding secondary antibodies for 12–16 h at 4°C. After a final three washes of 10 minutes each in 0.1 M PBS, the whole-mounted retinas were placed on a microscope slide with the GCL up and cover slipped with Fluoromount-G and nail polish was used to seal the coverslip.

**Table 1:** Antibodies used in the study.

Antibody	Antigen/ immunogen	Species, dilution	Source; catalog No.
**ChAT**	ChAT purified from human placenta	Goat polyclonal; 1:500	Millipore; AB144P
**CART**	Rat CART Peptide aa 55–102	Rabbit polyclonal; 1:2000	Phoenix Pharmaceuticals; H-003-62
**GABA**	GABA-bovine serum albumin conjugate	Rabbit polyclonal; 1:500	Sigma; A2052
**GFP**	Green fluorescent protein purified from *Aequorea victoria*	Goat polyclonal; 1:650	GeneTex; GTX26673
**GFP-Alexa 488**	Green fluorescent protein purified from *Aequorea victoria*	Rabbit polyclonal; 1:500	Life Technologies; A21311
**Nf-H (SMI-32)**	non-phosphorylated epitope on heavy molecular weight neurofilament H (200 kD)	mouse IgG1, clone SMI-32; 1:650	Abcam; AB73273
**NgR1**	recombinant mouse Nogo receptor C27-S447	Goat Polyclonal; 1:100	R&D Systems, AF1440
**PV**	E. coli-derived recombinant human Parvalbumin alpha	Sheep Polyclonal; 1:500	R&D Systems; AF5058
**RBPMS**	RBPMS_4-24_ with N-terminal cys; GGKAEKENTPSEANLQEEEVRC- KLH conjugate	Guinea Pig affinity purified polyclonal; 1:1000	Brecha Lab; GP15029-F PhosphoSolutions Inc.; 1832-RBPMS

### Antibodies

The dilutions of the primary antibodies are provided in Table 1. Secondary antibodies used in this study were Alexa-594 Donkey Anti-Mouse IgG, Alexa-594 Donkey Anti-Rabbit IgG, Alexa-594 Donkey Anti-Guinea Pig IgG, and Alexa-594 Donkey Anti-Goat IgG at 1:800 (Jackson ImmunoResearch Laboratories, West Grove, PA). As a negative control, the omission of the primary antibodies in the single or double labeling studies confirmed the elimination of specific labeling for all antibodies used.

### Fluorescent image acquisition

Immunostaining was evaluated using a Zeiss Laser Scanning Microscope (LSM) 710 (Carl Zeiss, Thornwood, NY; RRID: SciEx_11637) with a Zeiss Plan-Apochromat 20x/.08 NA or a Zeiss C-Apochromat 40x/1.2 NA water objective, or on a Nikon TE 2000 Confocal Microscope C90i with either 20x (NA = 0.3) or 40x (NA = 0.95) objectives controlled by NIS-AR software (Nikon Instruments, RRID: SCR_014329). Confocal images were analyzed using Image Browser v4 (Zeiss or ImageJ. Image contrast was only scaled linearly for the final images presented in the figures., and Magenta and green pseudo-color was applied using Adobe Photoshop (Adobe Systems, San Jose, CA) or ImageJ for red-green color blind readers.

## Results

To visualize the expression of NgR1 in the retina, we used the *ngr1* conditional mutant mouse line (*ngr1 flx*) [11]. In this strain, exon 2 of the *ngr1* gene is flanked by *loxP* sites. This exon contains the complete coding sequence of the mature NgR1 receptor. Excision by Cre recombinase abolishes the expression of NgR1 and initiates the expression of GFP from the *ngr1* gene locus. Thus, in mice harboring one recombined allele (*ngr1* Δ) and one wild-type (WT) allele (+) with the genotype, *ngr1* Δ/+, NgR1 is expressed from the WT allele (+), and GFP is expressed from the recombined conditional allele (Δ)under control of the *ngr1* promoter. These mice express GFP in cells that express NgR1. Although the projection pattern of RGCs in constitutive *ngr1* mutant mice (KO) is unremarkable [10], and these *ngr1* KO mice exhibit normal visual acuity [22], we examined mice heterozygous for the mutant *ngr1* Δ allele to preclude the absence of NgR1 from potentially altering retinal development or causing other changes in retinal circuitry. As GFP expression levels in these mice are not sufficient to permit direct visualization of intrinsic GFP fluorescence [11], we employed antibodies directed against GFP to detect the protein.

**Fig 1:**
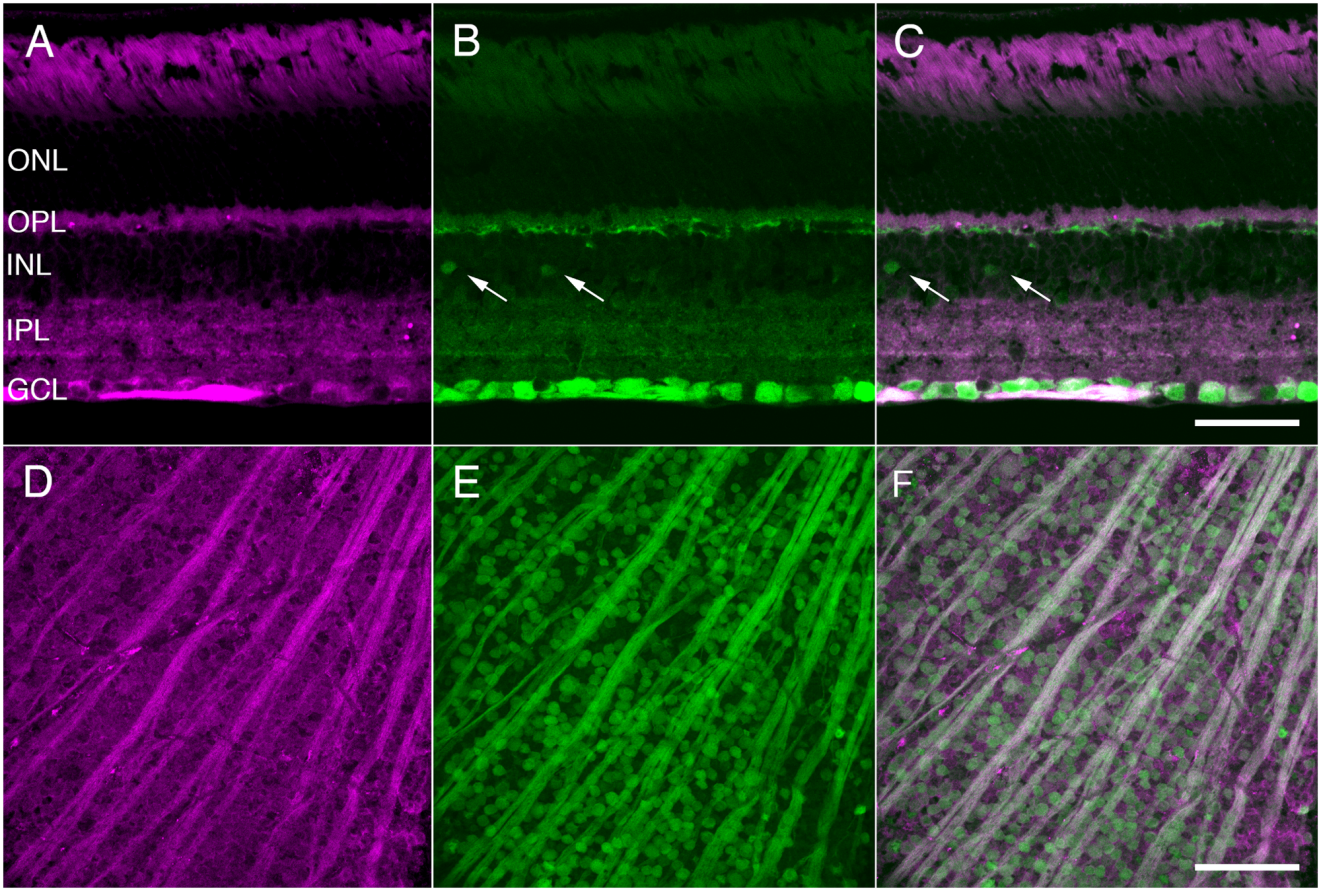
GFP expressed under the *ngr1* promoter identifies NgR1 expression. A-C. Vertical section from a *ngr1* Δ/+ mouse showing anti-NgR1 immunoreactivity (A, magenta), anti-GFP immunoreactivity (B, green) and a merge of the two images (C). GFP-positive somatas and processes are present in the INL (arrows). D-F. Whole-mounted retinas showing anti-NgR1 (D), anti-GFP (E) and merged (F) immunoreactivity in the GCL and RNFL. GCL, ganglion cell layer; INL, inner nuclear layer; IPL, inner plexiform layer; ONL, outer nuclear layer; OPL, outer plexiform layer. Scale bars: 100 μm.

To verify that GFP expression in this mouse line was consistent with NgR1 immunoreactivity, retinas from these *ngr1* Δ/+mice were immunostained with GFP and NgR1 antibodies (Fig 1). In retinal cross sections, NgR1 immunostaining was localized primarily to GCL somata and the retinal nerve fiber layer (RNFL), consistent with previous reports [27,30]. A band of weak immunoreactivity was also observed in strata 4 of the inner plexiform layer (IPL) as well as the outer plexiform layer (OPL), and nonspecific immunoreactivity was observed in the outer segments of the photoreceptors (Fig 1A). In comparison, GFP immunoreactivity in the GCL was localized to somata and the RNFL, similar to the pattern of NgR1 immunoreactivity in the GCL. Expression of GFP was present in a minority of somata in the inner nuclear layer (INL) (Fig 1B and 1C arrows), as well as fine processes in the OPL. These two features were not detectable with antibodies to NgR1 (Fig 1A), and thus we did not pursue them further. To verify that GFP expressing cells in the GCL and RNFL fibers contained NgR1 immunoreactivity, we analyzed these layers in whole-mounted retinas. GFP expressing somata and axons in the RNFL contained NgR1 immunoreactivity (Fig 1D–1F). Conversely, NgR1 immunoreactive somata also expressed GFP (Fig 1F).

**Fig 2:**
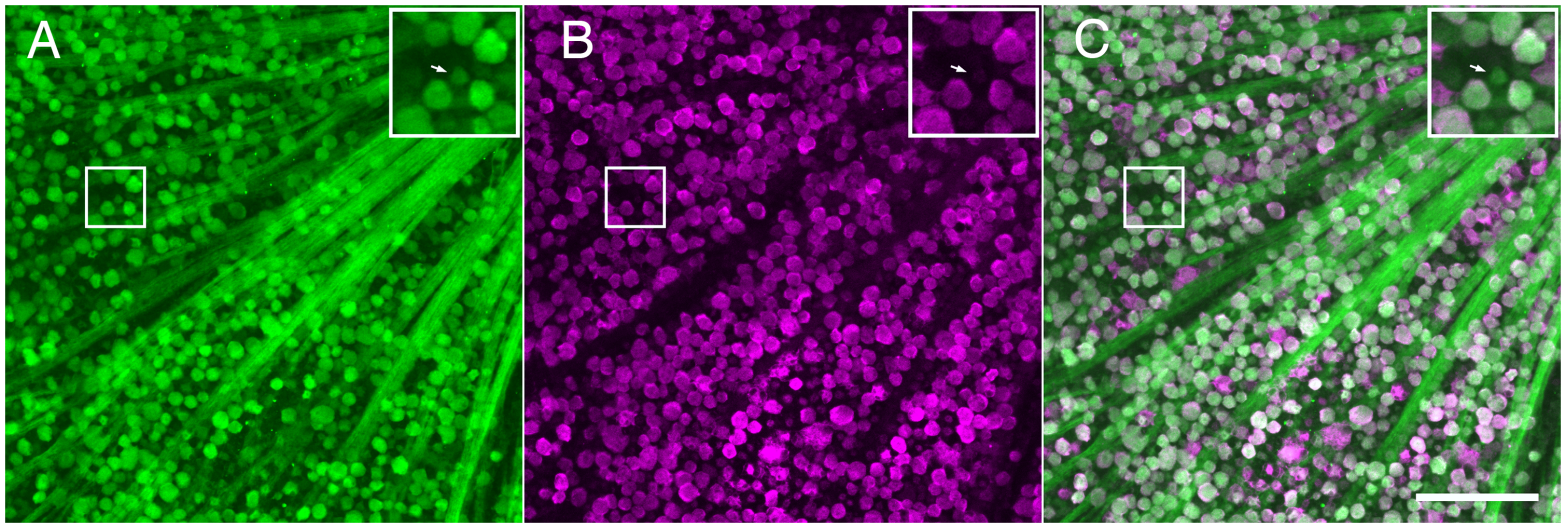
Comparison of anti-GFP and anti-RBPMS immunoreactivity in *ngr1* Δ/+ mice indicates NgR1 is expressed nearly ubiquitously among RGCs. A. Image of GCL and RNFL from whole mount retina showing anti-GFP immunoreactivity (green). Top right inset shows magnified view of boxed region; arrow points to GFP positive cell with a small soma relative to neighboring cells. B. Same field of view as in A, showing anti-RBPMS immunoreactivity (magenta). Top right inset shows magnified image of boxed region; arrow points to same cells as inset to A, which is negative for RBPMS immunoreactivity. C. Merge of images from A and B. Scale bar: 100 μm; insets are magnified 2X from boxed areas.

To determine if the GFP expressing cells in the GCL were RGCs, we examined whole-mounted retinas stained with antibodies directed against GFP and RBPMS (Fig 2). RBPMS is expressed in RGCs ubiquitously in the mouse retina while other identified RGCs markers such as Brn3a label only a subset of RGCs [35–37]. More than 99% percent of cells immunoreactive for RBPMS also exhibited GFP expression (39,727/40,043 cells from 3 retinas), indicating that nearly all RGCs express NgR1. There were also cells with specific immunoreactivity for GFP in the GCL for which RBPMS immunoreactivity was not detectable (Fig 2, insets). These cells exhibited smaller diameter somata, suggesting they may represent a population of displaced amacrine cells [34,38].

**Fig 3:**
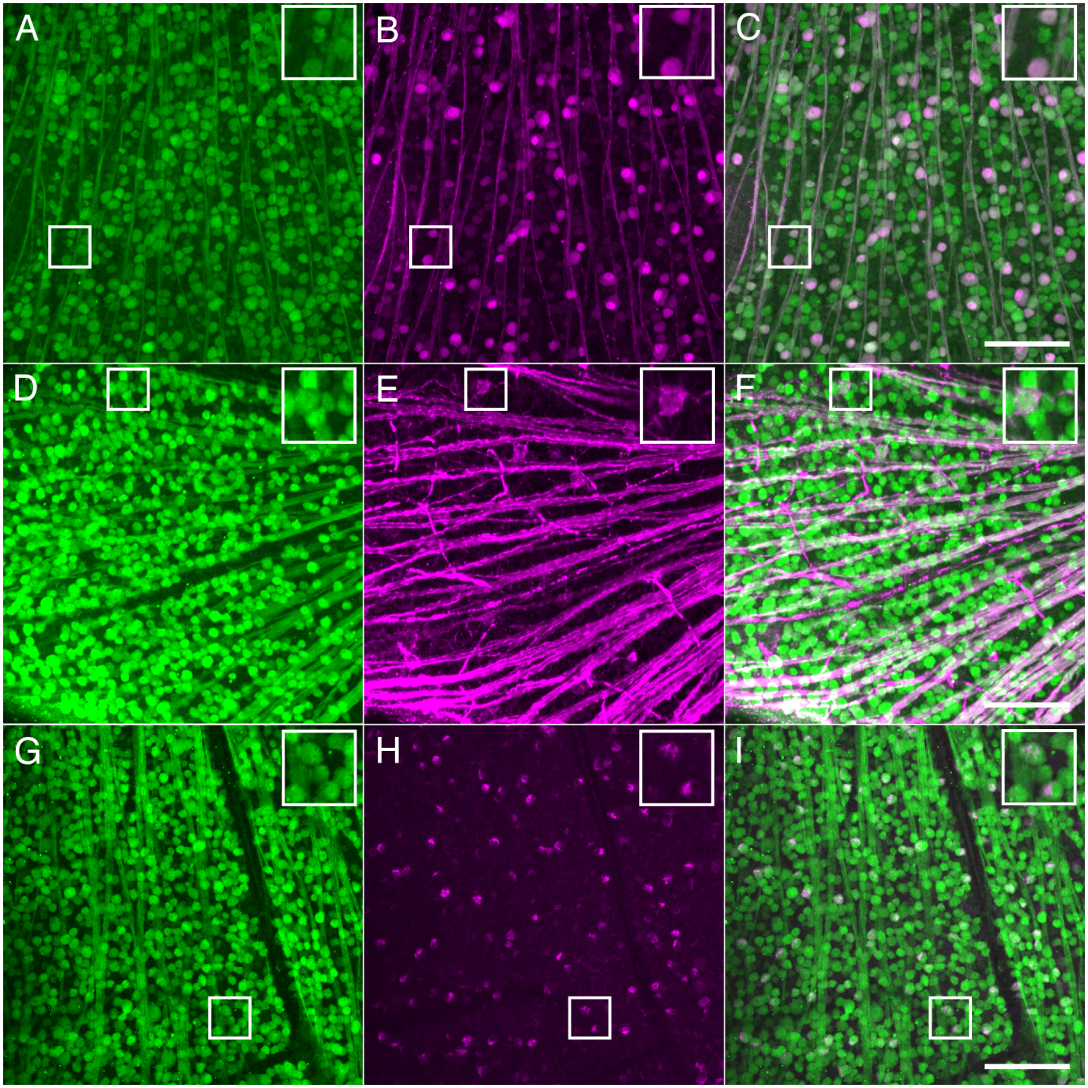
NgR1 expression by specific types of RGCs. A-C. Whole-mount view of GCL showing immunoreactivity in a *ngr1* Δ/+ mouse for anti-GFP (A), anti-PV (B), and a merge (C). D-F. Same as A-C, but showing anti-SMI-32 immunoreactivity (E, magenta). G-I, Same as A-C, but showing anti-CART immunoreactivity (H, magenta). Scale bar: 100 μm; insets are magnified 2X from boxed areas.

**Fig 4:**
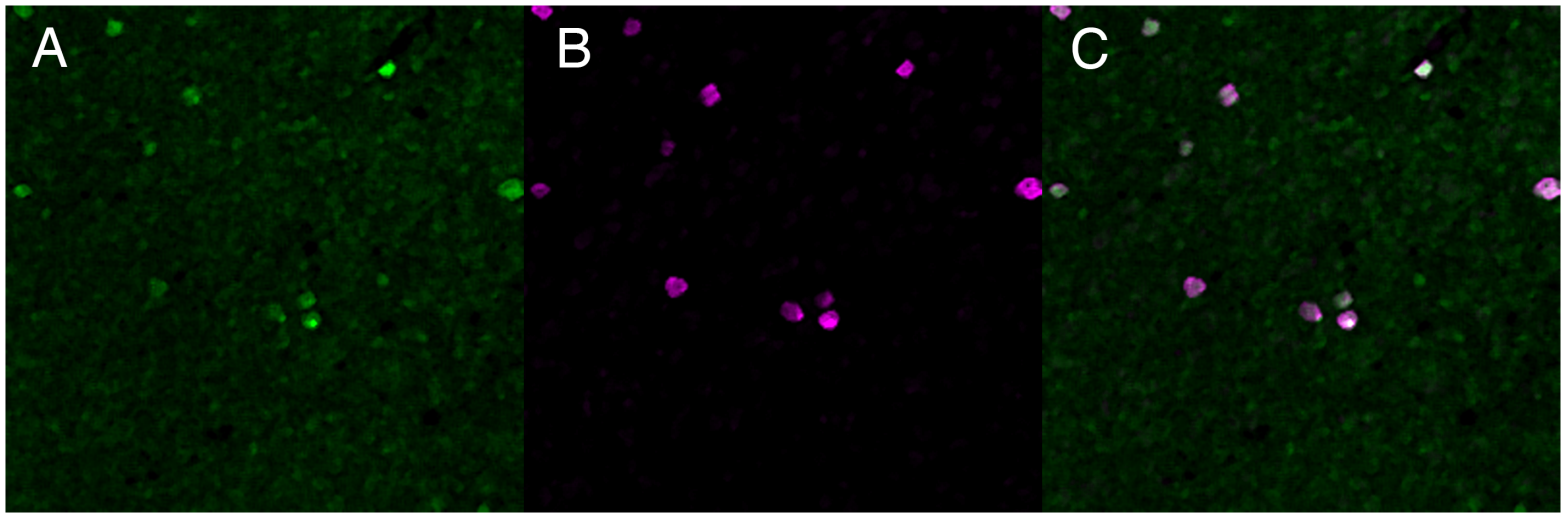
NgR1 expression by displaced RGCs in the INL. A-C. Whole-mount view of INL showing immunoreactivity in a *ngr1* Δ/+ mouse for anti-GFP (A), anti-RBPMS (B), and a merge (C). Scale bar = 50 μm.

To verify expression of NgR1 in multiple types of RGCs, we compared GFP expression with immunoreactivity of several RGC types including PV-expressing RGCs, ON-sustained and OFF-transient alpha RGCs, and ON-OFF directionally sensitive RGCs. PV is a calcium binding protein whose retinal expression is variable between species, but is expressed in large retinal ganglion cells in mice [39], and by approximately 29% of all RGCs [40]. PV-positive RGCs have been divided morphologically into eight distinct cell types [40]. We found that all PV immunoreactive cells also expressed GFP in the *ngr1* Δ/+mice, indicating this subclass of RGCs all contain NgR1 (Fig 3A–3C). We further examined two well-characterized RGC types: ON-sustained and OFF-transient alpha RGCs. These RGCs express non-phosphorylated neurofilament heavy chain recognized by the SMI-32 antibody [41]. All SMI-32 immunoreactive cells expressed GFP (Fig 3D–3F), confirming that these two populations of alpha RGCs express NgR1. In addition, the CART antibody labels ON-OFF direction-selective RGCs [42]. GFP was expressed in all RGCs immunoreactive for CART (Fig 3G–3I), indicating all ON-OFF direction-selective RGCs express NgR1. To explore whether sparse populations of RGCs might also express NgR1, we examined the co-expression of GFP and RBPMS by displaced RGCs in the INL. Many of these cells are likely intrinsically-photosensitive RGCs expressing melanopsin reside [43] (Fig 4). We found that displaced RGCs indicated by their immunoreactivity for RBPMS, were also immunoreactive for GFP. These experiments support the conclusion that NgR1 expression in RGCs is nearly ubiquitous.

**Fig 5:**
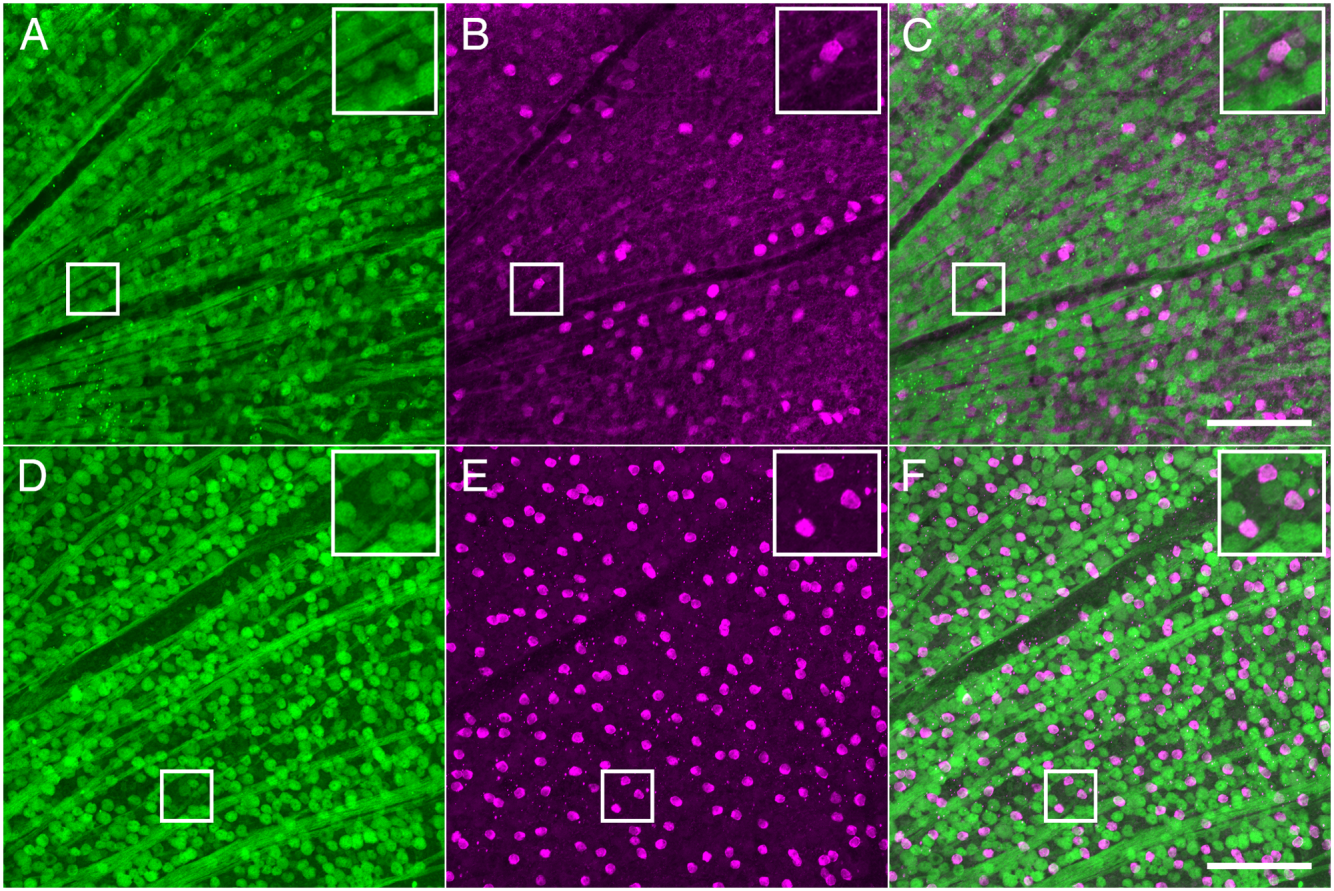
A subset of GABAergic and all cholinergic amacrine cells express NgR1. A-C. Whole-mount retina from *ngr1* Δ/+ mice showing immunoreactivity to anti-GFP (A, green), GABA (B, magenta), and a merged image (C). D-F. Same as A-C, but showing anti-ChAT immunoreactivity (E, magenta). Anti-ChAT in the GCL labels ON type starburst amacrine cells. Scale bar: 100 μm; insets are magnified 2X from boxed areas.

We also examined the smaller diameter cells in the GCL that were not immunoreactive for RBPMS (Fig 2, insets). Roughly half of the cells in the GCL are amacrine cells. While there are multiple types of amacrine cells, most can be identified as belonging to one of two broad classes: wide-field amacrine cells that express GABA or narrow-field amacrine cells that express glycine [44,45]. However, glycine immunoreactive amacrine cells have not been reported in the GCL. Thus, to confirm that these GFP-positive, RBPMS-negative cells were amacrine cells, we examined GABA immunoreactivity in *ngr1* Δ/+mice. A subset of GABA-immunoreactive cells, although not all of them, exhibited detectable anti-GFP immunoreactivity (Fig 5A–5C). One type of wide-field GABAergic amacrine cell that is known to be present in the GCL is the ON type starburst amacrine cell. These cells are also ChAT immunoreactive [46,47]. Thus, to test whether NgR1-positive amacrine cells in the GCL were starburst cells, we immunolabeled for ChAT and GFP in the *ngr1* Δ/+ mice. GFP immunoreactivity was detectable in ChAT immunoreactive cells, though the signal was systematically weaker than cells immunoreactive for RBPMS and other RGC markers. These experiments indicate that the GCL expression of NgR1 includes ON starburst amacrine cells.

## Discussion

We have used *ngr1* Δ/+ mice to determine the expression patterns of NgR1 in the GCL of the retina. We first established that these mice express GFP in NgR1 immunoreactive cells in the GCL, and then showed that these cells included both RGCs and displaced amacrine cells. Next, we established that all RGCs including well-characterized PV-positive, ON-OFF direction-selective, ON-sustained, and Off-transient alpha RGCs express NgR1. Last, we determined that a subset of GABAergic displaced amacrine cells also express NgR1, including ON-starburst amacrine cells. These results clarify that NgR1 is expressed not only by the vast majority of RGC types but a subset of displaced amacrine cells in the GCL. These observations are in general agreement with previous studies [27,30], but extend upon previous findings to demonstrate the presence of NgR1 in specific RGC types and demonstrate that NgR1 expression is not limited to RGCs. Our experiments were facilitated by the recent identification of the pan-RGC marker RBPMS [35], and the utilization of a transgenic mouse that expresses GFP from the *ngr1* gene locus [11]. Previous studies did not distinguish NgR1 expression in RGCs versus so-called displaced amacrine cells: these displaced amacrine cells represent a significant cell population in the GCL. Some displaced amacrine cells, including ON-starburst amacrine cells, express NgR1.

The presence of NgR1 in nearly all RGCs supports its potential as a therapeutic target for optic nerve damage. Recent experiments have demonstrated that NgR1 blockade can increase RGC axon regrowth as well as improve RGC survival in a variety of optic nerve pathologies, including glaucoma models, ischemic models, and following traumatic injury [27,28]. If NgR1 was selectively expressed on only some types of RGCs, this would limit the potential for complete recovery of vision. Furthermore, because different RGC types transmit distinct signals to ‘image forming’ and ‘non-image forming’ pathways, partial recovery of RGC function could have unintended consequences for vision, vestibular-ocular reflexes, circadian entrainment, and other system that receive retinal input (reviewed in [33,48]). On this note, while co-labeling with RBPMS and GFP reveals that the great majority of RGCs express NgR1, this approach does not confirm that NgR1 is expressed in every rare RGC type. However, the observation of RGCs displaced to the INL exhibiting GFP immunoreactivity in *ngr1 Δ/+ mice* is suggestive that M1 intrinsically photosensitive RGCs express NgR1 [49].

The expression of NgR1 by RGCs and along their axons is particularly interesting given the recent observation that Nogo-A is also expressed by RGCs and localizes to axons [50]. Whether Nogo-A expressed by RGCs contributes to regulating the growth, recovery, and plasticity of RGCs is unclear at present. Future studies of mutant mice lacking Nogo-A in specific RGC populations could be an avenue to test this possibility. Similarly, the retinal expression of Nogo-A and NgR1 invites further investigation into a possible retinal contribution to the recovery of visual acuity in models of amblyopia as NgR1 constitutive mutant mice exhibit a slow but near complete recovery of acuity following long-term monocular deprivation [22].

The expression of NgR1 by amacrine cells is intriguing given that these cells are the most morphologically diverse and least understood cell class in the retina (reviewed in [48]). The presence of NgR1 on a subset of starburst amacrine cells is interesting given that these interneurons change function during development [51]. At early time points (E-19 to P10) these cells are responsible for creating the excitatory waves that lead to correlated RGC activity and the subsequent topographical ordering of the central visual system [52–54]. Later, the primary role of these cells changes and they mediate the spatially asymmetric inhibition that shapes the tuning of direction-selective RGCs (reviewed in[47]). Given the requirement for NgR1 to close the critical period for ocular dominance plasticity in primary visual cortex, perhaps Nogo-A and NgR1 play a role in this developmental switch as well. It should also be noted that NgR1 expression was not observed in OFF starburst amacrine cells; the somata of these cells lie in inner nuclear layer. This observation of a differential pattern of NgR1 expression across amacrine cell types may indicate a role for NgR1 in the establishment and wiring of circuits within the retina.
